# From Threat to Challenge: Understanding the Impact of Historical Collective Trauma on Contemporary Intergroup Conflict

**DOI:** 10.1177/17456916221094540

**Published:** 2022-08-09

**Authors:** Mengyao Li, Bernhard Leidner, Gilad Hirschberger, Jiyoung Park

**Affiliations:** 1 Max Planck Institute for Research on Collective Goods; 2 School of Psychology, Queen’s University Belfast; 3 Department of Psychological and Brain Sciences, University of Massachusetts Amherst; 4 Baruch Ivcher School of Psychology, Reichman University; 5 Department of Psychology, University of Texas at Dallas

**Keywords:** collective trauma, intergroup conflict, threat and challenge, biopsychosocial model

## Abstract

Collective memories of trauma can have profound impact on the affected individuals and communities. In the context of intergroup conflict, in the present article, we propose a novel theoretical framework to understand the long-term impact of historical trauma on contemporary intergroup relations from both victim and perpetrator perspectives. Integrating past research on intergroup conflict and the biopsychosocial model of threat and challenge, we argue that people appraise their group’s past victimization and perpetration differently, either as a threat or as a challenge. Shaped by contextual factors and individual differences, these differential appraisals will subsequently influence how group members respond to contemporary intergroup conflict, with both adaptive and maladaptive consequences. This model contributes to unifying the previous research that has shown diverse effects of historical trauma on present-day intergroup dynamics. We present preliminary empirical evidence in support of the framework and discuss its theoretical and practical implications.

Collective trauma is an earth-shattering episode in a group’s history that has a profound, lasting impact not only on the people directly involved but also on entire communities and even on generations after the traumatic event (Bouchat et al., 2017; Hirschberger, 2018). Intergroup violence often inflicts collective trauma, for both victims and perpetrators—if in (very) different ways. For victims, collective suffering may promote a perpetual sense of fear and group vulnerability and increases their vigilance to signs of threat (Canetti et al., 2018). For perpetrators, collective harm doing poses a direct threat to the group’s moral image and identity (Shnabel et al., 2009), impairs group members’ health and well-being (Leidner et al., 2015), and may even lead to long-term posttraumatic reactions (Bracken et al., 1995).

A growing body of research has uncovered that the effects of collective trauma can extend from the past such that collective memories of harm that took place even before current group members were born continue to affect intergroup relations in the present day (Vollhardt & Bilewicz, 2013). In such cases, collectively transmitted trauma casts a long shadow that influences not only conflicts between groups that have been direct adversaries in the past (Rimé et al., 2015), but also conflicts removed in time and space from the original traumatic event (e.g., Canetti et al., 2018; Hirschberger et al., 2021; Schori-Eyal, Klar, & Ben-Ami, 2017; Schori-Eyal, Klar, Roccas, & McNeill, 2017).

Much of the literature on the long-term effects of historical collective trauma has been based on a view of trauma as a serious threat to the group that leads to maladaptive outcomes, especially in terms of creating psychological barriers to contemporary intergroup harmony (e.g., Canetti et al., 2018; Ein-Dor & Hirschberger, 2013; Lifton, 2005; Markiewicz & Sharvit, 2021; Vollhardt & Nair, 2018). A more scarce but growing body of research, however, has questioned the maladaptiveness of these outcomes (Hirschberger & Ein-Dor, 2020) and shown that collective trauma can also lead to constructive intergroup attitudes and behavior in present day (e.g., Rees et al., 2013; Vollhardt & Staub, 2011; Warner et al., 2014). Drawing on the biopsychosocial theory of threat and challenge (Blascovich & Mendes, 2000), in the current article, we propose a novel theoretical framework to unify these diverse understandings of the link between historical collective trauma and contemporary intergroup dynamics. We argue that group members’ divergent reactions to collective trauma can be understood using an appraisal framework that distinguishes between threat- and challenge-based appraisals of the traumatic event. Importantly, this framework allows for a more pluralistic view of the adaptiveness of the appraisals. Although threat-induced responses are often maladaptive for promoting peaceful conflict resolution and intergroup harmony, they can be adaptive for preserving the in-group and ensuring its security and survival (see also Hirschberger & Ein-Dor, 2020). Although challenge-induced responses are generally adaptive for promoting peace, they can be maladaptive when they are driven by perceived military strength and aimed at enhancing group power.

In an integrative model illustrated in Figure 1, we propose that group members appraise their historical collective trauma (victimization or perpetration) differently, either as a threat or a challenge to their group, according to their subjective assessment of the situational demands posed by the trauma relative to the resources that they perceive to have to effectively cope with the demands. Threat and challenge appraisals of historical trauma should then have differential implications for how people react to contemporary intergroup conflicts. We also argue that how people appraise historical trauma will be further influenced by both contextual (e.g., societal narrative, power dynamics in conflict) and individual-difference (e.g., in-group identification, attachment security) factors.

**Fig. 1.**
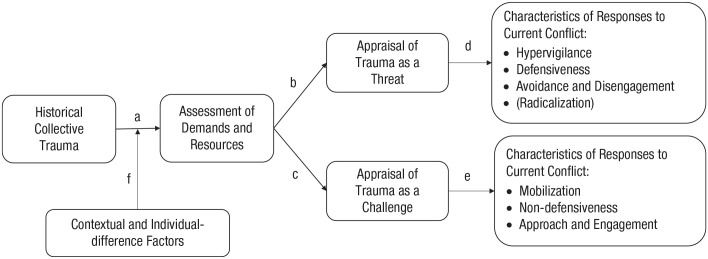
A general process model of collective trauma illustrating the pathway from historical collective trauma to group members’ responses to current intergroup conflict through appraisals of the past trauma as a threat or a challenge.

In the current article, we first conceptualize collective trauma as resulting from both collective victimization and collective perpetration (see also Bilali & Vollhardt, 2019; Hirschberger, 2018; Li & Leidner, 2019) and then review the threat-centered psychological discourse on the contemporary relevance of historical collective trauma with a focus on its impact on current intergroup conflicts. Next, we introduce the threat-challenge framework (Fig. 1), which is rooted in the biopsychosocial theory (Blascovich & Mendes, 2000) and offers a unified and pluralistic perspective on the long-term effects of collective trauma on intergroup conflicts. We further discuss examples of contextual and individual-difference factors as moderators of threat and challenge appraisals. Finally, we shed light on the ways in which the new framework can inspire future research on intergroup conflict and its practical implications.

## Collective Trauma: Victimization Versus Perpetration

Several different terms are used in the literature to refer to a traumatic collective past. The terms “collective victimization” and “collective victimhood” focus exclusively on the experience of victims and survivors, and “collective perpetration” and “collective harm doing” focus on the experience of perpetrators. Victim and perpetrator groups clearly have different experiences regarding the traumatic past, but they also have a certain shared collective memory of the event as well. To account for both the differences and similarities in perceptions of a traumatic past, we opted to use the overarching term “collective trauma,” which includes both collective victimization and perpetration and is defined as

a traumatic event that affects an entire society; it does not merely reflect an historical fact, the recollection of a terrible event that happened to a group of people. It suggests that the tragedy is represented in the collective memory of the group. (Hirschberger, 2018, p. 1)

We further emphasize that this definition of collective trauma differs from the definition of individual trauma in that collective trauma reflects the long-term social and political implications of a traumatic history that are transmitted across time and space and does not refer to individual psychopathological reactions to trauma that have received attention elsewhere (e.g., Bonanno, 2004). Thus, collective trauma includes the collective experiences of all sides involved, and we focus on the experience of collective trauma born out of suffering and out of perpetrating intergroup violence.

According to the needs-based model of reconciliation (Shnabel & Nadler, 2008), intergroup conflicts lead to asymmetrical identity losses among victim and perpetrator groups. Whereas victims experience losses of group agency in terms of power, status, and control, perpetrators experience losses of group moral image and identity (Shnabel & Nadler, 2008, 2015). Although it has been argued that only a small minority of victims and survivors develop posttraumatic stress disorder at the clinical level (Bonanno, 2004), collective victimization has been shown to negatively affect group members’ psychological well-being in a wide variety of social, political, and historical contexts. Collective suffering of war, political violence, repression, torture, and other mass atrocities can contribute to feelings of anxiety, fear, sadness, and distrust; perceived loss of control; avoidance behaviors; social isolation; and loneliness (Beristain, 2006; Lykes et al., 2007; Zwi & Ugalde, 1989). Such impact can even be transgenerational, with second- and third-generation survivors also exhibiting posttraumatic symptoms that are attributable to older generations’ exposure to collective victimization (Yehuda et al., 2001). More pertinent to the current theorization, these individual consequences of trauma can also manifest at the collective level such that the trauma becomes part of the group’s psyche (e.g., national psyche), contributes to the creation of a collective narrative, and serves as a prism through which current affairs are perceived and understood (Alexander et al., 2004; Lifton, 2005). Intergroup transgression and violence can also take a toll on members of the perpetrator group (Leidner et al., 2015; Li & Leidner, 2019; Shnabel & Nadler, 2008). Similar to collective victimization, collective perpetration can also be experienced as a form of trauma with adverse effects on group members’ health and psychological well-being at the individual level (for a review, see Leidner et al., 2015) and shape the group psyche among both immediate and distant survivors of trauma at the collective level (Imhoff et al., 2013; Lifton, 2005). Unlike collective victimization, however, the lingering impact of collective perpetration manifests primarily in struggles with the group’s challenged moral values, norms, and reputation (Shnabel & Nadler, 2008), which are often accompanied by feelings of collective shame and guilt (Lickel et al., 2005), or efforts to justify or disengage from the transgression (Giner-Sorolla et al., 2011).

This research implies that individuals can experience collective trauma not only by directly or vicariously suffering intergroup harm but also by directly or vicariously committing intergroup harm. In the following section, we review in detail the existing literature that has adopted a threat-centered framework to understand the long-term impact of historical trauma on contemporary intergroup relations from both victim and perpetrator perspectives.

## Collective Trauma From the Past to the Present: A Threat-Centered Perspective

Despite the abundance of research on the short- and long-term effects of collective trauma primarily on victims and survivors, less attention has been paid to its ripple effects on new intergroup situations that are seemingly unrelated to the original traumatic event. The Holocaust, for example, has created a lasting, intergenerational, collective trauma among both Jews and Germans, albeit in very different ways (Giesen, 2004; Hirschberger et al., 2022; Imhoff et al., 2017; Kellermann, 2001). It is a central component of Jewish as well as German identity (e.g., Fulbrook, 1999; Pew Research Center, 2013) and serves as a lens through which both groups perceive the contemporary world and understand the ongoing relationship between themselves and other groups (e.g., Canetti et al., 2018; Hirschberger, 2018; Hirschberger et al., 2022; Schori-Eyal, Klar, & Ben-Ami, 2017; Schori-Eyal, Klar, Roccas, & McNeill, 2017; Vollhardt, 2012). As Lifton (2005) contended, following collective trauma, the pains and suffering of past events often become indistinguishable from current conflicts.

### Threats of collective victimhood

A great deal of social-psychological work has been devoted to understanding the multitude of threats caused by victimization. In addition to identity threat caused by loss of agency (Shnabel & Nadler, 2008), victim-group members can also experience threats to autonomy (Kachanoff et al., 2020), dignity and self-respect (Jogdand et al., 2020), and value and meaning (Hirschberger, Ein-Dor, et al., 2016). In cases of extreme intergroup violence, collective victimization can also entail an existential threat that promotes a perpetual sense of group vulnerability, collective angst, and a mindset of being “an expiring people, forever on the verge of ceasing to be” (Rawidowicz, 1967, p. 423; Hirschberger & Ein-Dor, 2020; Hirschberger, Ein-Dor, et al., 2016; Kelman, 1992; Schori-Eyal, Klar, & Ben-Ami, 2017; Schori-Eyal, Klar, Roccas, & McNeill, 2017; Wohl et al., 2010).

Victimhood has been typically viewed as a conflict-supportive belief and as having negative consequences for intergroup relations (Markiewicz & Sharvit, 2021; Vollhardt & Nair, 2018). Only a small minority of research has also shown prosocial intergroup outcomes in the aftermath of victimization (Vollhardt & Bilali, 2015; Vollhardt & Staub, 2011), and such prosocial tendencies are often conditional (Warner et al., 2014). Groups that have survived extreme violence tend to view current intergroup conflicts as a continuation of the historical trauma and are highly suspicious of out-groups (Hirschberger & Ein-Dor, 2020). For example, when reminded of historical trauma from past collective victimization, Jewish Israelis who had a perpetual in-group victimization orientation increased attributions of malevolent intentions to out-group members in ambiguous situations in the present (Schori-Eyal, Klar, & Ben-Ami, 2017). Likewise, after being reminded of an exclusive framing of the Holocaust (“the Holocaust is a crime against the Jewish people”) compared with inclusive framings (“the Holocaust is a crime against humanity”), Israelis increased support for aggressive policies regarding Iran and the Palestinians (Canetti et al., 2018). When such a reminder was juxtaposed with criticism from international allies, even left-wing Israelis, who typically support peaceful policies, changed their opinion and increased their support for political militancy (Hirschberger et al., 2017). In a different intergroup context, Americans who were reminded of their group’s historical victimization (e.g., Japanese attack on Pearl Harbor) experienced less collective guilt for their group’s harm doing in Iraq over 70 years later (Wohl & Branscombe, 2008). These reactions are deemed maladaptive when examined from an intergroup-relations perspective. When considering them from a group-protective perspective (Hirschberger & Ein-Dor, 2020), however, reacting to reminders of collective trauma with increased vigilance and defensiveness is at times adaptive because it may promote the safety and security of the group.

### Threats of collective perpetration

Collective perpetration has primarily been understood as representing a threat to the moral identity of the group (Bilewicz & Stefaniak, 2013; Branscombe & Doosje, 2004; Leidner & Castano, 2012; Leidner et al., 2010; Shnabel et al., 2009), and the descendants of perpetrators are motivated to restore the group’s moral image (Shnabel & Nadler, 2008). Perpetrator groups may contend with their group’s past transgressions by experiencing in-group-critical moral emotions and exhibiting more prosocial intergroup behavior as a form of moral improvement (Brown et al., 2008; Gausel et al., 2012; Gausel & Leach, 2011; Mashuri et al., 2018; Rees et al., 2013). But more often than not, group members react defensively to reminders of collective harm doing (for similar arguments, see Bilali, 2013; Bilali et al., 2019; Li & Leidner, 2019). Such reactions can range from adopting strategies to disengage from the immorality of the group’s actions (Bandura, 1999; Castano & Giner-Sorolla, 2006; Leidner et al., 2010), for example, by making defensive attributions that attempt to exonerate the group (Hirschberger, Kende, et al., 2016) or by attributing the crimes committed to external causes (Doosje & Branscombe, 2003; Imhoff et al., 2017), to distancing themselves from the wrongdoing (Li et al., 2021; Peetz et al., 2010) to actively moralizing the harm committed (Giner-Sorolla et al., 2011; Leidner & Castano, 2012) to resisting future efforts to restore justice and peace (Leidner et al., 2010; Li et al., 2020) and to claiming competitive victimhood (Sullivan et al., 2012).

Although only a handful of prior studies have directly examined the impact of historical collective harm doing on responses to contemporary conflicts, the existing literature suggests that reminders of past harm doing and a threat-dominated perception of the past might lead to excessive in-group defense when group members are confronted with current conflicts. Research on the contemporary relevance of historical wars provides indirect evidence supporting this argument, showing that reminders of one’s country’s engagement in a historical interstate war increased citizens’ support for future violence against countries uninvolved in the original war (Li et al., 2016). Note that such an increase in support for future violence was explained by heightened generalized perceptions of threat from foreign countries unrelated to the historical trauma.

The literature reviewed above highlights that a group’s traumatic history may elicit overly defensive reactions to present conflicts in subsequent generations of both victim and perpetrator groups, especially when this history is viewed in monolithic and simplistic terms that are predicated on threat. Subsequent generations of victims tend to become more defensive when they see the trauma as evidence that the world is against them (Bar-Tal & Antebi, 1992a, 1992b) or does not share in their suffering (Vollhardt, 2013; Wohl & Branscombe, 2005), when they essentialize the perpetrator group and view them as inherently evil (Campbell & Vollhardt, 2014), or when they perceive current threats as indistinguishable from past traumas (Canetti et al., 2018; Lifton, 2005). Subsequent generations of perpetrators tend to become more defensive when the trauma, posing an unbearable threat to the moral image and identity of the group, feeds into defensive representations of history (Hirschberger, Kende, & Weinstein, 2016).

On the one hand, the view of trauma as catastrophic and threatening, often eliciting excessive in-group defense in the face of new conflicts, represents the perspectives and experiences of many trauma descendants in the world. On the other hand, it paints a simplified, incomplete picture of how past traumatic events can affect individuals and groups in present day. By integrating the previous literature on the diverse intergroup outcomes of collective trauma, we propose that a more pluralistic, comprehensive outlook on the long-term impact of collective trauma should consider both the threat and the challenge that it poses as well as both its maladaptive and adaptive consequences for in-group security and intergroup relations.

## Beyond Threat: A Threat-and-Challenge Framework of Collective Trauma

Collective memory of trauma is not merely the recollection of a historical event; it is the outcome of an appraisal process by which individuals give meaning to the historical event (Leach, 2020). For the victim group, one important aspect of meaning making in trauma is whether group members perceive themselves as standing alone against the rest of the world and suffering more than other groups (i.e., siege mentality: Bar-Tal & Antebi, 1992a, 1992b; or exclusive and competitive victimhood: Noor et al., 2012; Vollhardt, 2009b) or whether they can see the nuances in their relationship with other groups during the traumatic event and recognize allies and helpers among perpetrators. For the perpetrator group, meaning making in collective harm doing could involve the appraisal of the in-group’s action as implicating morally flawed aspects of the group’s nature or specific aspects of the group’s behavior that can be controlled and rectified (thereby resulting in collective shame vs. collective guilt, respectively; Lickel et al., 2005, 2011). These differential appraisals could explain why some members of victim groups and perpetrator groups react to the traumatic past as a threat, whereas others may see it as a challenge or opportunity.

When Jan Tomasz Gross (2001) published *Neighbors*, which described the burning of the Jews of Jedwabne in a barn by their Polish neighbors, many Poles reacted with rage because this episode violated their need to believe in Poland as a nation victimized by the Nazis (Vollhardt et al., 2015). Shevach Weiss, the Israeli ambassador to Poland at the time, however, said that he also remembered other barns—referring to the Poles who hid him and other Jews in their barn during WWII. This “other barn” narrative, which includes both the horrific crimes and the acts of grace that took place during the traumatic past, does not just provide a more complex understanding of history; it provides another prism through which to understand current intergroup dynamics. Likewise, the 2015 terror attacks on Paris can be seen simply as a Muslim attack on Europe or the West, but the other-barn perspective allows one to see that the security guard at the Stade de France who blocked a suicide bomber from entering the stadium was a Muslim, and so were the thousands who denounced the attacks.

From an appraisal perspective (Leach, 2020), the other-barn metaphor illustrates how group members may come to make sense of group behavior. In this particular example, the behavior of the perpetrator group can be appraised either as indicating an inherent internal evil in the group or as including both the horrific and the honorable acts during the ominous past. The other-barn perspective thus allows group members to recognize the nuances and complexities of group behavior and helps transform a dark and threatening episode in intergroup relations into an opportunity that could potentially ameliorate these relations. For members of the victim group, such a perspective allows them to view the world not through the narrow lens of exclusive and perpetual victimhood but through the more inclusive lens of interconnectedness and solidarity with other groups, even including some members of the perpetrator group. For the perpetrator group, the other-barn perspective makes it harder to attribute the harm doing to the nature of their group that is beyond one’s control and instead makes room for responsibility taking and acts of moral repair (Gausel et al., 2012; Lickel et al., 2005).

### The biopsychosocial model of threat and challenge

Our distinction of perceptions of trauma as threat versus challenge is grounded in the biopsychosocial theory of threat and challenge (Blascovich & Mendes, 2000; Mendes et al., 2002; Tomaka et al., 1997). According to this theory, threat and challenge are two distinct motivational states that individuals may experience while engaging in motivated-performance situations that require active cognitive responses, which result from evaluations of situational demands relative to available coping resources. A threat response occurs when perceived demands exceed perceived resources, whereas a challenge response occurs when perceived resources exceed perceived demands. In addition to factors directly modulating task engagement during motivated-performance contexts (e.g., task difficulty, self-relevance or goal relevance), a wide variety of other factors can also modulate the appraisal processes. For example, situational demands can be determined on the basis of psychological and physical danger, uncertainty, and required effort in a given situation, whereas the amount of personal resources can be assessed on the basis of skills, knowledge, abilities, and other dispositional characteristics of an individual as well as the availability of external support (Blascovich, 2008; Blascovich et al., 2002).

These differential appraisals of situational demands vis-à-vis resources result in distinct profiles of behavioral, affective, cognitive, and physiological responses that are either activational (characteristic of challenge) or inhibitional (characteristic of threat). In particular, challenge-and-threat states can be differentiated with cardiovascular reactivity (i.e., cardiac output and total peripheral resistance) while individuals engage in active tasks that are self-relevant or goal relevant. Threat states are characterized by decreased heart efficiency and vasoconstriction, whereas challenge states are characterized by greater heart efficiency and vasodilation (e.g., Blascovich et al., 2004; Mendes et al., 2008). The theory further contends that challenge states generally lead to more adaptive outcomes, such as better task performance, because they motivate approach-oriented coping, energy mobilization, and greater task engagement (e.g., Blascovich, 2008; Tomaka & Blascovich, 1994). Threat responses, by contrast, are relatively more variable, fragile, and stressful that often manifest as heightened vigilance, avoidance, and task disengagement (e.g., Blascovich, 2008; Blascovich et al., 2002). Although threat responses are usually considered less adaptive, some of these responses may not necessarily be maladaptive and may in fact be highly adaptive in certain situations that require hypervigilance and avoidance because these states can help protect and preserve the resources necessary for survival (Blascovich, 2008; Tomaka & Blascovich, 1994).

The biopsychosocial theory has been applied to examine individual and group differences in stress and emotion in a variety of contexts. For example, threat states and challenge states have been shown to occur, respectively, when people interact with others who belong to a different rather than the same racial group (Blascovich et al., 2001) or others who are atypical rather than typical members of their group (Mendes et al., 2007), when they receive negative rather than positive social feedback (Mendes et al., 2007), and when they perceive themselves to be in a position of low rather than high power (Akinola & Mendes, 2014; Scheepers et al., 2012).

Drawing on this theory, we argue that appraisals of collective trauma map onto the key distinctions made between appraisals of stressors as a threat or a challenge. Going back to the other-barn metaphor, this perspective does not view history only as a continuous threat against the group but looks for the silver lining even in the direst of circumstances. Historical trauma can also present resources such as allyship, solidarity, and moral courage. When perceived resources outweigh perceived demands, trauma can be appraised as a challenge that both victim and perpetrator groups can overcome. In the next section, we integrate the previously disconnected literatures on biopsychosocial theory and intergroup violence and propose a novel framework to understand divergent appraisals of historical trauma and their implications for responses to contemporary intergroup conflict.

### The threat-and-challenge model of collective trauma

#### Demands and resources

Our conceptual model (Fig. 1) proposes that perceptions and appraisals of collective trauma are determined according to assessments of demands and resources among the affected (victim and perpetrator) groups (Fig. 1, Path a; Table 1). On the one hand, historical trauma presents a variety of situational demands. For the victim group, collective victimization can serve as a reminder of loss of agency, dignity, and autonomy; physical and symbolic harm to the in-group; and even danger to the group’s very existence and survival (Hirschberger & Ein-Dor, 2020; Hirschberger, Ein-Dor, et al., 2016; Jogdand et al., 2020; Kachanoff et al., 2020; Shnabel & Nadler, 2008). For the perpetrator group, collective harm doing can serve as a reminder of loss of moral identity and reputation as well as social exclusion from the broader society and the international community (Shnabel & Nadler, 2008). Such demands can be further heightened by punishment, sanctions, and other formal measures of retributive and compensatory justice imposed on the perpetrator group (Wenzel et al., 2008). Regardless of the in-group’s victim or perpetrator status, collective trauma caused by intergroup violence should highlight issues of safety and security for the in-group.

**Table 1.** Potential Demands and Resources Relevant to Victim, Perpetrator, or Both Groups (List Not Exhaustive)

	Victim group	Perpetrator group	Shared
Demands	• Loss of agency, dignity, autonomy • Physical and symbolic harm • Danger to group’s existence	• Loss of moral identity and reputation • Social exclusion • Retributive and compensatory justice	• Physical danger • Insecurity
Resources	• Moral identity • Group cohesion, solidarity, and efficacy • Allies and friends • Retributive and compensatory justice	• Agency aspects of identity • Controllability of group behavior • Opportunities to repair	• Values and beliefs • Meaning making • Restorative justice

On the other hand, reminders of a historical trauma may also make available resources of the group salient. Because the moral aspects of victims’ identity are intact (Shnabel & Nadler, 2008), they can serve as powerful psychological resources for victim-group members to cope with the trauma. Additional resources for the victim group might include group cohesion, solidarity, and collective efficacy even in the face of adversity (Bandura, 2000; Drury et al., 2019); allies and friends who joined their struggle against the perpetrators (Reicher et al., 2006); and retributive- and compensatory-justice measures (e.g., David & Choi, 2005; Li et al., 2018). For the perpetrator group, in contrast, the agency aspects of their identity can be a potential resource given that they suffer from the loss of the moral rather than agency aspects of social identity (Shnabel & Nadler, 2008). Relatedly, perceived controllability of group behavior (e.g., when highlighted by the morally exceptional individuals who stood in solidarity with the victims) and opportunities to repair the harm doing can also serve as resources that help perpetrator-group members overcome the dark episode of their group’s history. Like demands, there are likely shared resources for both victim and perpetrator groups, which may include “lessons learned” that result in core group values and beliefs (e.g., “we shall overcome,” “God is with us,” “Never again”; Klar et al., 2013), meaning making (Hirschberger, 2018), and restorative approaches to justice that seek to reaffirm previously violated values and restore relations between groups (e.g., Wenzel et al., 2008). Table 1 displays examples of demands and resources in the context of collective trauma that might be relevant to victim, perpetrator, or both groups.

Depending on the overall relative assessments of such demands and resources, we argue, members of the same (victim or perpetrator) group can have different appraisals of collective trauma, viewing it either as a threat or as a challenge to their group (Fig. 1, Paths b and c). Note that our model does not propose specific combinations of perceived demands and resources that could lead to a threat or a challenge appraisal. In addition to the examples listed in Table 1, individuals can perceive a wide range of idiosyncratic demands and resources, influenced by both contextual and individual-difference factors (as discussed in more detail below). According to Blascovich et al. (2002), individuals simultaneously consider all elements of demand and resource as well as “their additive and synergistic effects” (p. 91). In a given situation, however, it is possible that one of these elements becomes particularly salient and triggers a high overall threat or challenge appraisal (Blascovich et al., 2002). In keeping with the biopsychosocial theory, we maintain that the overall ratio of perceived demands over resources will determine the ultimate threat or challenge appraisal. Below, we hypothesize the general behavioral responses to contemporary intergroup conflicts as a result of threat and challenge appraisals of historical collective trauma among victim and perpetrator groups. We also provide examples of more specific response patterns likely triggered by specific demands or resources.

#### Threat appraisals of collective trauma

The central tenet of our theoretical model is that threat and challenge appraisals of historical collective trauma will have downstream implications for how people respond to contemporary intergroup conflict. In line with the biopsychosocial model of challenge and threat, when faced with a current conflict, individuals’ threat appraisals of historical trauma should primarily motivate behavioral responses characterized by heightened vigilance, avoidance, and disengagement (Fig. 1, Path d).

##### Collective victimization

For victim-group members, we argue, threat-induced responses to current intergroup conflict should mainly aim at protecting the in-group and ensuring its safety and survival. When victim-group members perceive an overwhelming danger to the existence and survival of the in-group, for example, they may respond by avoiding or moving away from any conflict situation and adversarial group that could potentially pose an existential threat that they are unable to cope with (Hirschberger & Ein-Dor, 2020). Such avoidance-oriented response could also entail disengagement from both antisocial and prosocial actions in contemporary intergroup situations. Indeed, perceived threat because of a loss of power can result in an *inertia effect*, a general behavioral tendency toward inaction (Ginges & Atran, 2008). In a series of studies, Ginges and Atran (2008) showed that Palestinians who experienced humiliation (conceptualized as an emotional outcome of losing group power) in their conflict with Israel reduced support for both political violence and political compromise, suggesting an overall inertia effect. Although this research did not examine a carryover effect from old to new conflicts, it is reasonable to speculate that such a tendency toward inaction might extend beyond the original conflict and shape reactions to other conflicts with other groups as well.

Many of the threat-induced responses among victims resemble those induced by in-group-defensive victim beliefs that have been previously examined (e.g., exclusive and competitive victimhood, siege mentality, perpetual-victimhood orientation; Bar-Tal & Antebi, 1992a, 1992b; Noor et al., 2012; Schori-Eyal, Klar, & Ben-Ami, 2017; Schori-Eyal, Klar, Roccas, & McNeill, 2017; Vollhardt & Bilali, 2015). The current framework, however, extends the existing literature by emphasizing perceived coping potential in determining the behavioral response to present-day conflicts. In other words, although beliefs about the in-group’s unique, exclusive, or perpetual victim experiences highlight tremendous situational demands, whether these beliefs would translate into threat-oriented behavior in new conflicts also depends on the victims’ assessment of the resources available to the group.

##### Collective perpetration

For perpetrator-group members, threat-induced responses should mainly aim at defending the in-group’s morality and may manifest as moral disengagement, denial, and avoidance when dealing with a current conflict. When the threat appraisal is based on perceived moral loss that cannot be restored, group members might resign from any attempt to repair broken relations with other groups and instead engage in psychological distancing, cover-ups, or other forms of withdrawal to mitigate the perceived moral threat. This hypothesis is coherent with the conceptualization of collective shame as an emotional response to perceived global, innate moral flaws of the in-group leading to avoidance behavior, especially when moral repair is deemed impossible (Lickel et al., 2011). Other research has offered a more nuanced view of the appraisal basis of collective shame, distinguishing between shame resulted from perceived damage to the in-group’s public reputation (i.e., image shame) and perceived violation of its core moral values (i.e., moral shame; Allpress et al., 2014). In two different intergroup contexts—the Holocaust and the Iraq War—Rees et al. (2013) found that among German and British participants, feelings of image shame but not moral shame for the in-group’s past war transgressions predicted social distancing from contemporary immigrants (i.e., Turks in Germany and Pakistani in the United Kingdom). These findings suggest that not all demands triggered by past collective perpetration would lead to threat-oriented responses to new intergroup conflicts. These demands vary in their relevance, intensity, and the resources required to cope with them and hence have divergent implications for contemporary intergroup dynamics. Although a threat mindset is most frequently associated with avoidance-oriented responses, it can also trigger processes related to approach motivation by activating alternative goals that could be approached (Nash et al., 2011). Such approach-oriented reactions can help relieve the anxious uncertainty associated with perceived threat. For example, group members can turn to ideological extremism and engage in radical actions in an attempt to defend the group against perceived threat (McGregor et al., 2012). We therefore argue that threat appraisals could also lead to approach-motivated reactions to intergroup conflicts, such as radicalization and extreme violence.

For both victim and perpetrator groups, their threat-induced responses may be considered maladaptive from an intergroup-relations perspective because they are unlikely to be conducive to peaceful resolution of intergroup conflict. However, the threat mindset does not always entail maladaptive outcomes; rather, they can at times be beneficial for the survival of the in-group (Hirschberger & Ein-Dor, 2020). For example, whereas avoidance can be viewed as a maladaptive coping strategy at the individual level in the context of trauma (Berman et al., 1996; Dempsey, 2002), recent work has challenged this view and shown its adaptive functions, such as preventing harm to the self (Hofmann & Hay, 2018). At the collective level, both hypervigilance and avoidance can function as group-protective mechanisms (Hirschberger & Ein-Dor, 2020). Avoiding a war that the group cannot win, for example, can indeed be considered adaptive for the in-group. For victim-group members, a perpetual state of hypervigilance as a result of historical trauma and (indirect) experiences of life-threatening events is psychologically taxing, but at the same time, it serves to enhance alertness to potential dangers to the in-group, thereby protecting the individual or the group from reexperiencing the same trauma in the future. For perpetrator-group members, avoiding or disengaging from in-group-committed harm is maladaptive from a conflict-resolution perspective but can be adaptive from a group- and self-protective perspective because it preserves the moral image of the in-group and, by association, of the self.

### Challenge appraisals of collective trauma

As discussed earlier, collective trauma also presents opportunities for both victim and perpetrator groups to proactively cope with and overcome the trauma. This alternative perspective is consistent with challenge appraisals of trauma (Fig. 1, Path c). Challenge-induced responses to contemporary conflicts, we propose, should be characterized by action mobilization, nondefensiveness, and approach-oriented tendencies (Fig. 1, Path e).

#### Collective victimization

In the context of suffering intergroup violence, past victimization has been shown to promote positive personal and collective transformations, such as empathy, prosocial behaviors, and a sense of responsibility for others (“altruism born of suffering”; Staub & Vollhardt, 2008; Vollhardt, 2009a). For example, people who experienced interpersonal or group-based harm exhibited increased empathy and decreased in-group bias, resulting in greater willingness to help out-groups in need (Vollhardt & Staub, 2011). Likewise, Jewish participants who considered the lessons learned from the Holocaust felt more morally obliged to help other victimized groups unrelated to their suffering (e.g., Sudanese and Chinese). Such perceived moral obligation, however, did not extend to current adversaries of the in-group (e.g., Palestinians; Warner et al., 2014, Study 2).

Although these past studies did not look at perceived resources as motivating prosocial responses to current conflicts, such responses are particularly in line with a challenge mindset rooted in morality-related resources. Perceived moral stance and moral identity of their group, we argue, may not only enable victims to overcome hardships of the past but may also empower them to respond prosocially to present intergroup conflicts. Other types of coping resources—for example, perceived group cohesion, solidarity, and efficacy—can promote collective resilience (Drury et al., 2019) and collective action to advance peace and justice (van Zomeren et al., 2012). In addition to social support and solidarity in one’s own group, perceived solidarity across group boundaries—in particular, a shared victim identity with other groups—can also foster conciliatory intergroup attitudes and peace activism (Adelman et al., 2016; Shnabel et al., 2018; Vollhardt, 2009b, 2015; Vollhardt & Bilali, 2015). Our model suggests that such inclusive victim beliefs can help enable a challenge mindset that motivates historical victim groups to participate proactively in peace processes when faced with contemporary conflicts.

Our recent research offers direct support for some of these propositions. In one study, Jewish Israelis responded to historical in-group trauma (i.e., the Holocaust, compared with an out-group trauma—the Nanjing Massacre—or no trauma) with a stronger pattern of cardiovascular challenge (vs. threat) responses (Kretchner et al., 2022). Note that this effect was observed only among Jewish Israelis who felt psychologically stable and secure. Among these individuals, their cardiovascular reactivity characteristic of challenge was associated with greater self-reported moral obligation to help other victims of intergroup violence and was indirectly related to greater support for peacemaking with Palestinians. These results indicate that the memory of collective victimization does not inadvertently lead to greater threat-induced responses in contemporary intergroup conflict, as much of the literature suggests (e.g., Hirschberger et al., 2017; Schori-Eyal, Klar, & Ben-Ami, 2017), but that this response depends on the ability to perceive coping resources and reappraise the trauma as a challenge.

#### Collective perpetration

In the context of collective perpetration, research has shown that rather than defensively avoiding the unpleasant repercussions of in-group-committed violence and deflecting blame, people at times actively engage with the harm doing by confronting specific deviant in-group members who are responsible (Castano et al., 2002; Marques et al., 2001), by confronting the in-group as a whole and protesting against its actions (Iyer et al., 2007; Leach et al., 2006; Packer, 2008, 2009; Packer & Chasteen, 2010; Packer & Miners, 2012), or by demanding justice for out-group victims (Leidner et al., 2010). The proposed model suggests that these proactive, nondefensive responses are likely the behavioral outcomes of a challenge mindset, enabled by perceived resources such as group agency and the ability and opportunity to control, repair, and improve group behavior. As discussed earlier, perceived controllability of group behavior is a core cognitive basis of collective guilt, which has been shown to be associated with approach tendencies and moral repair (Lickel et al., 2011). Such a challenge mindset could therefore motivate members of historical perpetrator groups to engage in more tolerant behaviors toward out-groups in contemporary intergroup conflicts.

In our research directly based on the biopsychosocial model (McLamore et al., 2021), we found that Americans’ cardiovascular responses consistent with challenge (relative to threat) predicted peaceful and prosocial responses to reminders of past in-group perpetration, such as less psychological defensiveness and greater support for diplomacy. This effect was observed only among individuals in the in-group-trauma condition, in which participants were reminded of intergroup violence committed by their own group against another group, and not in the condition describing intergroup violence between two different out-groups.

As discussed above, challenge responses are typically understood as adaptive for the individual and the in-group and can also foster positive intergroup relations. One exception to the adaptiveness of challenge appraisals is a challenge mindset predicated on perceived hard power—military capacity, in particular. This challenge mindset might drive people to support and engage in intergroup violence, with a primary goal to strengthen the in-group rather than to resolve the conflict peacefully. Note that although both threat and challenge appraisals can motivate aggressive responses to present conflicts, the underlying motivations are distinct: Whereas threat-induced violence is driven by the motivation to defend the in-group and ensure its survival, challenge-induced violence is driven by the motivation to enhance the in-group and capitalize on opportunities to gain resources or to preserve its superiority. In sum, both threat- and challenge-oriented responses can be adaptive or maladaptive, depending on the sources of demands and resources and whether the ultimate goal is in-group protection/enhancement or peaceful conflict resolution.

## Contextual and Individual-Difference Moderators

### Contextual factors

Our proposed process model considers contextual and individual-difference factors that determine appraisals of demands and resources (Fig. 1, Path f). Examples of contextual factors include the type and magnitude of demands that a particular traumatic event poses, objective resources available to the group or the individual to deal with a conflict, societal narratives and discourses, and the power dynamics between groups. This list is far from exhaustive, and discussing every one of these factors is beyond the scope of the current contribution. Below, we focus on two critical contextual factors that have been widely studied and/or discussed in past research and scholarship: societal narrative and power dynamics.

#### Societal narrative

Distinct from individual trauma, collective trauma by definition carries a wider societal dimension and can be understood only within the specific historical and sociopolitical context. Societies often go through stages to make sense of and deal with the trauma, resulting in various collective narratives and discourses that are transmitted across generations. These collective narratives provide shared interpretations of the traumatic history, which shape how group members come to understand the trauma and act in present-day intergroup situations (Bilali & Vollhardt, 2019; Taylor et al., 2020). Historical perpetrator groups, for example, may share a collective narrative that capitalizes on the group’s victim rather than perpetrator experiences (e.g., Serbs in the aftermath of the Yugoslav wars; Taylor et al., 2020). The presence and saliency of collective narratives also shift in response to societal and political changes (Markiewicz & Sharvit, 2021). Interviews of individuals directly affected by the 2020 Nagorno-Karabakh war revealed that narratives around old trauma such as the Armenian genocide and the Khojaly massacre were frequently brought up by both sides to justify the use of violence during the war (Sotieva, 2021). Societal narratives therefore play a paramount role in shaping both people’s appraisal of collective trauma and their behavior in contemporary conflicts.

#### Power dynamics

Research has distinguished between symmetrical and asymmetrical conflicts, characterized by equal and unequal power relations, respectively (e.g., Kteily et al., 2013; Penić et al., 2021). In asymmetrical conflicts, members of the high-power group might be less likely to see the demands of the situation outstrip the group’s resources. Members of the low-power group, in contrast, might not have the necessary resources to help them overcome the situational demands, especially if they are still suffering from the adversarial impact of the historical trauma, such as poverty and structural violence. This is not to suggest, however, that high-power groups have an inherent advantage over low-power groups in terms of appraising the traumatic event as a challenge. As we propose in the present model, power is only one of the many dimensions of demand and resource potentially relevant to the groups affected by collective trauma. People can still appraise the trauma as a threat if they perceive an overall higher ratio of demands over resources. In situations in which perceived power is salient enough to trigger an overall challenge appraisal, the implications of such appraisal for present-day conflict can still depend on whether perceived power is mainly concerned with “soft power” (e.g., political values, culture, institutions; Nye, 1990) or “hard power” (e.g., military capability). Whereas the former is likely to lead to nondefensive or even prosocial responses, the latter is likely to lead to engagement in violence.

The 9/11 terrorist attacks brought home a strong sense of collective victimhood to Americans, who are also citizens of the world’s biggest economic and military power. This unique combination of victimhood and high-power status makes the United States an intriguing case for understanding the role of power in the long-term intergroup implications of collective trauma. In the two decades following the attacks, Americans engaged in many different “survivor missions” (Lifton, 2005). According to Lifton (2005), feelings of humiliation and victimization mobilized many Americans to support the official survivor mission—the “war on terror”—against countries with no clear connection to the 9/11 attacks. One key element, however, is missing from this analysis—the tremendous military power of the United States. Whereas collective trauma typically diminishes a sense of power among members of the victim group, the strong objective (hard) power of the group might buffer victims against such a demand and instead enable them to adopt a challenge mindset and to engage in aggressive “survivor missions.” By contrast, feelings of humiliation should be more likely to translate into inertia or inaction among low-power victim groups (Ginges & Atran, 2008; or turn to ideological extremism, Lindner, 2001). Considering power dynamics as a contextual factor can therefore help researchers understand why different victim groups can take on vastly different actions in contemporary conflicts.

### Individual-difference factors

Previous research has identified various individual-difference factors as moderators of motivational states of challenge and threat in different interpersonal and social contexts. For example, people with high (rather than low) self-esteem (Seery et al., 2004) or strong (rather than weak) belief in a just world (Tomaka & Blascovich, 1994) are more likely to appraise novel stressors as a challenge rather than a threat. Thus, not all people appraise a stressor in the same way. Likewise, we argue that not all members of a certain group will appraise their historical collective trauma in the same way. Some are more likely to appraise historical trauma as a threat, whereas others are more likely to appraise it as a challenge. In fact, some people may not engage in such appraisals at all if the traumatic event bears little relevance or significance to them (Leach, 2020). This is perhaps even more likely, and therefore important, for distant or secondary victims and perpetrators who are by default more removed, both physically and psychologically, from the original traumatic event or events. Individuals who view their group’s past as a closed chapter (i.e., historical closure; Hanke et al., 2013) or their group as consisting only of the current generation (Kahn et al., 2017), for example, might be unlikely to perceive collective trauma transmitted from previous generations as relevant enough to warrant further appraisal. Although this initial stage of the appraisal process is not explicitly considered in our model, it is a prerequisite for interpreting and coping with collective trauma.

To illustrate the individual differences that potentially shape threat or challenge appraisals, we focus on a group-level factor and an individual-level factor: (a) group identification (in-group glorification vs. in-group attachment) and (b) attachment security. We chose these factors because both involve a similar psychological resource—a sense of attachment—that may enable people to transform a painful and threatening history into an opportunity, a challenge. Attachment at the group level conveys a sense of belongingness to a social, national, religious, or ethnic community and to a cultural worldview, and this sense of belongingness may effectively buffer anxiety (e.g., Greenberg, 2012). At the individual level, attachment reflects a sense of ontological security: the notion that others are trustworthy, have goodwill, and will be present to help at times of threat. The sense of attachment security functions as a resilience factor in the face of various threats (Mikulincer & Shaver, 2016).

#### Group level: in-group glorification versus attachment

Substantial research has demonstrated that responses to intergroup violence depend on the type and strength of people’s identification with their group. The bidimensional model of attachment and glorification (Roccas et al., 2006, 2008) has been proposed to provide a unifying understanding of two broad facets of group identification: attachment and glorification. Whereas attachment is characterized by perceived importance of belonging to the group and commitment to it, glorification is characterized by beliefs in the in-group’s superiority over other groups and deference and unconditional loyalty and submission to in-group norms, traditions, and authorities. According to Cichocka (2016), glorification reflects an insecure type of in-group positivity (i.e., collective narcissism), which triggers hypersensitivity to threats and defensive reactions aimed at protecting the in-group. Indeed, considerable research has shown that glorification is associated with the use of in-group-defensive strategies (e.g., moral disengagement, dehumanization of out-group perpetrators) in response to in-group-committed violence (Bilali, 2012; Leidner & Castano, 2012; Leidner et al., 2010; Roccas et al., 2006) and in-group-suffered violence (Li et al., 2018). Pertinent to the current analysis, when reminded of the in-group’s engagement in a historical interstate war, individuals who strongly glorified their national in-group developed a heightened sense of intergroup threat, which generalized to out-groups that were unrelated to the original war (Li et al., 2016). Such generalized perceptions of threat further predicted support for aggressive approaches to dealing with contemporary international conflicts. Thus, glorification might predispose group members to be particularly sensitive to anything that can be interpreted as a threat to the in-group and hence are more likely to perceive high levels of “demands.”

By contrast, being attached to one’s group without glorifying it can be understood as a secure type of in-group positivity. Using the current framework, attachment seems to function as a psychological resource that provides a sense of nondefensive belongingness. Thus, attachment to the in-group negatively predicts the use of defensive strategies such as moral disengagement and dehumanization of out-group perpetrators (Leidner & Castano, 2012; Roccas et al., 2006) or is unrelated to them (Leidner et al., 2010; Li et al., 2018). Thus, in-group attachment (without glorification) might enable group members to appraise collective trauma as a challenge rather than a threat.

#### Individual level: attachment security

Individual differences in group identification may reflect a deeper sense of ontological security that is captured in one’s sense of attachment security. Because attachment theory is in essence a theory of individual and social defense against threat (Bowlby, 1982; Ein-Dor & Hirschberger, 2016), it may be particularly well suited for the study of challenge responses and threat responses to collective trauma.

According to attachment theory (Bowlby, 1973, 1980, 1982), the attachment-behavioral system modulates human responses to threat. Attachment security is the product of positive interactions with close others during early development, which over time fosters psychological stability and resilience and sustains mental health in adulthood (for a review, see Mikulincer & Shaver, 2016). Insecurely attached individuals, who have a history of more precarious interactions with others, may be more susceptible to threat and may respond to out-groups with adversarial reactions and a greater willingness to sacrifice for the in-group (Caspi-Berkowitz et al., 2019; Mikulincer & Florian, 2000; Mikulincer & Shaver, 2001, 2016). In contrast, research has shown that securely attached individuals are better able to cope with severe threats, such as military conflict (Grady et al., 2018; Mikulincer et al., 1993, 1999) or the terror of death (Mikulincer & Florian, 1998, 2000), and often transform threatening situations into opportunities for personal and interpersonal growth (Mikulincer et al., 2003).

We therefore suggest that individual differences in attachment security may modulate responses to collective trauma. Whereas insecure individuals are likely to exhibit threat responses such as defense of the in-group and hostility toward other groups, securely attached individuals may be more likely to transcend the threat, understand their trauma in inclusive terms (Vollhardt, 2009a), experience greater moral obligations to other victims (Warner et al., 2014), and exhibit an overall challenge response. We recently obtained some evidence for this proposition in a study examining cardiovascular and attitudinal effects following exposure to in-group historical trauma. In this study, participants high in attachment security that were exposed to footage of in-group trauma compared with out-group trauma or a control condition exhibited higher moral obligation toward victims and greater support for peacemaking with adversarial out-groups (Kretchner et al., 2022).

## Limitations and Future Directions

### Duality of conflict identities

Although our framework distinguishes clearly between the perspectives of victim groups and perpetrator groups, they are not mutually exclusive, clear-cut categories. Even in the same conflict, groups can experience both victimization and perpetration, albeit to different degrees (Bilali & Vollhardt, 2019; Li & Leidner, 2019; Vollhardt & Bilewicz, 2013). We therefore caution that the propositions regarding the experiences, appraisals, and behavioral responses of victim- or perpetrator-group members might not easily generalize to individuals with a dual identity (i.e., feeling both victim and perpetrator; SimanTov-Nachlieli & Shnabel, 2014). It seems plausible that “duals” can perceive a wider range of demands and resources than individuals with a clear victim or perpetrator identity, and the ultimate threat appraisal and challenge appraisal will depend on the availability, magnitude, and intensity of the specific demands and resources. Indeed, past research has shown that duals have the needs to restore both agency and moral identity (SimanTov-Nachlieli & Shnabel, 2014). In the same research, however, the need for agency took primacy in determining subsequent behavior toward the adversaries. Using the threat-and-challenge framework, one may hypothesize that the demands of victimhood can outweigh the demands of perpetration in determining the behavioral responses to new conflicts among people with a dual identity. Applying the threat-challenge framework to understand the perceptions, appraisals, and behavior of duals in conflicts will therefore generate fruitful theoretical development and empirical research.

### Temporal dynamics of threat appraisals and challenge appraisals

So far, we have discussed threat appraisals and challenge appraisals of collective trauma as an “either-or” psychological state. In reality, however, appraisal and coping are dynamic and continuous processes that respond to changing demands and resources over time (Leach, 2020; van Zomeren et al., 2012). In the current theorizing, we focus on a specific case of collective trauma—historical collective trauma that is transmitted across time, space, and generations. One unique feature of historical trauma is that people’s perceptions and appraisals of the trauma are constantly shaped and reshaped by the changing political, societal, and cultural contexts over time. The proposed model is therefore better understood as a dynamic one, involving continuous reappraisals of historical trauma in response to the changing circumstances and momentary saliency of different demands and resources.

It seems plausible, for example, that group members are more likely to appraise a traumatic event as a threat when it happened in the recent history and is still fresh in the collective memory. As time distances people from their traumatic past, they might perceive the demands of the trauma as less pressing and less intense and hence be more open to perceiving resources to cope with the past. To complicate this issue further, subjective perceptions of time can also be an outcome of threat appraisals or challenge appraisals. Past research has shown that compared with victims, perpetrators tend to perceive the same traumatic event as more distant in time (Li et al., 2021). Using the current proposed framework, subjective temporal distancing among perpetrators can be seen as an avoidance coping mechanism resulted from a threat appraisal based on perceptions of overwhelming demands (i.e., loss of morality). Future research can thus benefit from exploring the dynamic nature of the proposed model by, for example, conducting longitudinal studies in the aftermath of mass violence or comparing the responses of different generations following collective trauma.

### The interplay between historical and contemporary conflicts

Another crucial factor to consider when understanding the impact of past trauma on current conflicts is the characteristics of the current conflicts. Take the issue of power dynamics as an example. As discussed earlier, the relations between victim and perpetrator groups are often characterized by asymmetrical power dynamics. Historically victimized, low-power groups, however, can become the high-power group in an asymmetrical contemporary conflict (e.g., Klar et al., 2020). The role of the new power dynamic and the present-day perpetration (if any) by the historically victimized group should also be considered when trying to understand how historical victimhood shapes responses to current intergroup conflicts.

The resemblance between the past trauma and the present intergroup situation is another contextual factor that could moderate how appraisals of the past shape intergroup dynamics at the present. Research on altruism born of suffering showed that victims of collective trauma were more likely to exhibit prosocial behavior toward out-group victims who had suffered in a similar way, compared with out-group victims who had suffered in less similar ways (Vollhardt & Staub, 2011). For the perpetrator group, experiencing collective shame for the in-group’s past transgressions predicted attitudes toward contemporary intergroup relations more strongly when there was greater similarity between the current out-group and the previously harmed out-group (Rees et al., 2013, Study 2).

Taken together, we caution that although the current article offers a useful framework that systemizes the understanding of the various long-term effects of historical collective trauma on current conflicts, it should not be taken as providing an explanation for all responses to collective trauma or all the nuanced ways in which trauma can affect current conflicts. The proposed model focuses on explaining two broad categories of responses to current conflicts, one characterized by threat (i.e., hypervigilance, defensiveness, avoidance, and sometimes violent radicalization) and one characterized by challenge (i.e., mobilization, nondefensiveness, and approach). Future research should not only examine the processes leading to threat versus challenge responses but also the more nuanced variations within each type of response (e.g., threat-induced avoidance vs. radicalization).

## Contributions and Implications

The current article contributes to the literature on intergroup violence and conflict in several major ways. First, it provides an integrative, overarching framework that delineates the psychological processes linking collective trauma from the past to intergroup conflicts in the present. The threat-and-challenge model offers a common language to unify the previously disparate literatures, which have revealed group members’ divergent reactions to collective trauma that may seem inconsistent or even contradictory. Second, by conceptualizing collective trauma as a result of both collective victimization and perpetration, it considers both victim and perpetrator perspectives in tandem and therefore extends and integrates the existing intergroup literature that has mostly focused on one side of the conflict (cf. Bilali & Vollhardt, 2019; Li & Leidner, 2019; Shnabel & Nadler, 2008). Third, the proposed model places group members’ subjective assessment and interpretation of collective trauma at the core of understanding its long-term impact. In doing so, it emphasizes the agency of individuals even in the face of extreme violence and hardships. Finally, the current framework highlights a pluralistic account of the impact of collectively transmitted trauma by considering it as presenting both a threat and a challenge and having both adaptive and maladaptive outcomes. Our model therefore offers a road map to navigate the complexity of collective trauma.

The threat-and-challenge model of collective trauma also points to the importance of studying trauma, group-based violence, and conflict from a biopsychosocial perspective that allows multilayer assessments of their underlying mechanisms. Specifically, rather than relying on self-report measures alone, the motivational states of challenge and threat in intergroup situations may also be assessed and differentiated by physiological measures (i.e., cardiovascular responses). At a broader level, this suggests that physiological measures may complement self-report measures by capturing psychological processes that might exist outside of conscious awareness and therefore not easily accessible by introspection.

However threatening and devastating collective trauma may be, a rather simplistic view of group history may not only limit understanding of how groups recuperate and heal from the traumatic past but also obstruct efforts to restore the well-functioning of society. A complex and nuanced representation of a traumatic history has important implications for the meaning-making processes among descendants of historical victims and perpetrators, who must contend with the long-term consequences of the historical event. By considering its maladaptive and adaptive aspects, the threat-challenge perspective of collective trauma also has practical implications for in-group security and survival, conflict resolution, and peace building. Most importantly, this perspective suggests that collective trauma is not deterministic in its outcome. The fact that a group has suffered trauma through its history, be it as victim or perpetrator of intergroup violence, can lead to the continued suffering of trauma for generations to come—but it does not have to be this way. In the context of intergroup conflict, the proposed threat-challenge framework highlights the diversity in possible responses to collective trauma. By shifting from a threat to a challenge mindset, both victim- and perpetrator-group members can transform the past trauma not only to ensure its safety and survival but also to facilitate peaceful conflict resolution and positive intergroup dynamics when faced with contemporary conflicts.

## Transparency

*Action Editor:* Adam Cohen

*Editor:* Laura A. King
